# VistaScan: Optimizing Internet-Wide Scanning Through Visibility-Aware Distributed Task Allocation

**DOI:** 10.3390/s26051458

**Published:** 2026-02-26

**Authors:** Luolin Hu, Fan Shi, Yi Shen, Chengxi Xu, Pengfei Xue, Bingyang Guo

**Affiliations:** College of Electronic Engineering, National University of Defense Technology, Hefei 230031, China; huluolin14@nudt.edu.cn (L.H.); shenyi@nudt.edu.cn (Y.S.); xuchengxi@nudt.edu.cn (C.X.); xuepengfei@nudt.edu.cn (P.X.); guobingyang@nudt.edu.cn (B.G.)

**Keywords:** internet measurement, distributed scanning, node visibility, visibility matrix, visibility consistency, task allocation, network architecture

## Abstract

Internet-wide scanning is indispensable for security research and network measurement, yet its efficacy remains limited by significant visibility heterogeneity across global networks. Traditional centralized scanners suffer from single-point failures and offer a constrained perspective, while naive distributed approaches fail to intelligently leverage visibility variations, leading to redundant effort and incomplete coverage. This paper presents VistaScan, a novel distributed scanning system based on node visibility awareness, demonstrating that the visibility pattern among IP addresses is highly consistent within CIDR blocks, enabling a lightweight method for efficient and scalable quantification of per-node visibility. It first constructs a Visibility Matrix through efficient anchor probing, then employs a load-aware task allocation mechanism that assigns each block to the most capable node while filtering out entirely invisible blocks. Evaluation across global, regional, and challenging Special-Block tasks demonstrates that VistaScan consistently outperforms five baseline methods. It achieves near-optimal coverage (97.95%, 99.05%, and 97.58%, respectively), reduces probe volume by 64–93%, and shortens completion time by 13–19× compared to conventional centralized and distributed scanners. Furthermore, the visibility matrix derived from one port (TCP/80) effectively generalizes to other TCP ports (TCP/22, TCP/53), achieving coverages of 91.09% and 87.95%—preliminarily validating the practical generalizability of our approach. VistaScan provides both a highly efficient solution for Internet-scale distributed measurement and a new theoretical foundation based on visibility consistency, advancing the field from brute-force probing toward intelligent, low-overhead, and sustainable scanning practices.

## 1. Introduction

With the continuous proliferation of devices connected to the Internet, ensuring robust cybersecurity has become an imperative [1]. Internet-wide scanning plays an important role in cybersecurity analysis and network asset detection. This measurement technique has been used to study security vulnerabilities [2,3,4,5], host liveness [6,7,8,9], service deployment [10,11,12,13,14,15,16], network topology [17,18], uncover protocol weaknesses [19,20,21], and more. Internet-wide scanning works by initiating network connections with services on a set of given ports. So open port scanning is universally recognized as the critical first step in various types of network research. However, the scale of modern IPv4 and IPv6 networks makes exhaustive scanning a monumental task. Although modern scanning tools like ZMap [22], Masscan [23] and Nmap [24] employ centralized high-speed scanning from a single vantage point, this approach faces inherent limitations: the limited efficiency of a single node [12], network access restrictions [3,25] and IP blacklisting [9]. The research community has also recognized the fundamental constraint that no single node can see everything, and different geographical and network locations offer different views of the Internet [3,25,26,27,28].

To reduce the time overhead of large-scale scanning and detection, distributed technology was introduced in scanning and detecting network assets [29,30,31,32]. They improve probing efficiency by breaking down large-scale scanning tasks and distributing them across multiple nodes for execution. However, their task allocation strategies are often simplistic, treating different subtasks and probing nodes as interchangeable, and fail to account for the critical issue of nodal visibility divergence. Blindly assigning a CIDR block to any random node often results in insufficient coverage. If we missed certain ports from the very beginning, this omission can have significant negative implications for subsequent research, potentially resulting in the neglect of security vulnerabilities, the omission of critical assets, and a misjudgment of the overall security posture. Therefore, a more intelligent task allocation strategy is required to maximize coverage and minimize completion time.

In this paper, we present VistaScan, an intelligent visibility-aware distributed scanner for Internet-wide scanning. VistaScan is the first system that quantifies probing node visibility and, accordingly, performs task allocation and probing, the core idea of which is to assign probing tasks of different network blocks to the best-suited probing nodes. Our solution stems from a key empirical observation: Although nodes differ vastly in their global visibility (heterogeneity across nodes), a single node’s visibility of IPs within a specific CIDR block is highly consistent (consistency within blocks), which we formally define as the Visibility Consistency. Based on this, the visibility of representative anchor IPs by a node can characterize its visibility of the entire block. This allows for the assessment of a node’s capability without the need for a full-block exhaustive probe. Prior to executing the distributed probing, each node first performs a pre-scan of the representative IPs. The visibility scores of each node for each block are then calculated to construct a visibility matrix. Subsequently, the probing tasks are allocated in a targeted manner based on this matrix. This approach improves coverage effectiveness, reduces the total number of probe packets, shortens the overall scanning duration, and improves load balance in a distributed scanning scenario. Our core contributions are as follows.

- The first formalization and large-scale validation of Visibility Consistency. We discover and formally characterize the phenomenon that visibility remains consistent within blocks, enabling the use of a few representative IPs to infer whole-block visibility. We formulate this concept using Jaccard similarity and empirically establish /23 blocks as the optimal task allocation unit.
- A lightweight method for efficient and scalable quantification of per-node visibility. We show that using three anchor IPs from different probing sources is a practical and effective heuristic for characterizing block-level visibility. We also propose two anchor selection methods and construct a node–CIDR visibility matrix, where each value in the continuous range [0,1] reflects a node’s proficiency in probing a block.
- The design and implementation of the VistaScan system. VistaScan comprises four modules: CIDR Partitioning, Anchor Selection, Visibility Matrix Construction, and Load-Aware Task Allocation, significantly improving the performance of distributed Internet measurement by streamlining evaluation, quantifying capability, optimizing assignment, and pruning infeasible work.

We evaluated VistaScan with five baseline methods (Centralized, Union-of-All Nodes, Naive-Distributed-Random, Partitioned-Distributed-Random, Geo-Heuristic) in four metrics (Global Coverage, Total Completion Time, Total Packets Sent, Node Load Balance) across three scanning scenarios (global, regional, and challenging special block tasks). The results show that it achieves near-optimal coverage (97.95%, 99.05%, and 97.58% respectively) while reducing the total number of probes by 64–95% and overall completion time by 13-19x compared to centralized and naive distributed baselines. Crucially, we demonstrate that the visibility matrix derived from one port (TCP/80) is highly effective for scanning other TCP ports (TCP/22, TCP/53), achieving 91.09% and 87.95% coverage, which preliminarily underscores the generalizability of our approach.

The remainder of the paper is organized as follows. Section 2 details the background and motivation for this project. Section 3 describes the architecture of VistaScan. Section 4 reports the experimental setup and evaluation results, followed by a discussion of the results and limitations in Section 5. Section 6 presents the related work. Finally, Section 7 concludes this paper.

## 2. Background and Motivation

Distributed scanning leverages multiple geographically or topologically distinct vantage points to conduct large-scale network measurements in a parallel and coordinated manner. It offers increased scanning throughput, improved resistance to blocking, and, most importantly, diverse network perspectives. Compared to single-source methods, this approach significantly reduces scan time while enhancing fault tolerance and scalability. However, the transition to a distributed model replaces the technical challenge of high-speed packet generation with new algorithmic challenges, such as intelligent task allocation and coordination overhead. We begin by briefly summarizing current Internet-wide scanning systems and the key challenges they face.

### 2.1. Heterogeneity in Node Visibility

Prior studies have identified discrepancies in scanning results arising from different probing sources [3,25,26,27]. The paper [28] further showed that scanning from a single vantage point achieve limited coverage (miss an average 1.6–8.4% of HTTP, 1.5–4.6% of HTTPS, and 8.3–18.2% of SSH hosts) and that aggressive scanners experience significant blocking. Due to permanent and temporary blocking, probing nodes from different geographical and network locations would obtain different views of the Internet. They advise scanning from 2 to 3 sufficiently diverse vantage points to improve coverage, but this comes at the cost of a two-to-threefold increase in probing overhead.

To better understand the impact of nodal visibility on distributed probing, we designed a global-scale scanning experiment based on the MaxMind GeoLite2 database. We constructed a task list comprising 100 randomly selected CIDR blocks from each of 18 countries on six continents (excluding Antarctica) and performed scans from five geographically distributed nodes using ZMap. Each node operated at a calibrated rate of 1500 packets per second (pps), which is established in Appendix B to balance packet loss and resource usage. To reduce temporal bias, all nodes shared the same ZMap seed, ensuring target probing at approximately the same time. The experiment was repeated three times to mitigate the effects of the transient network, and the results were aggregated through a union between repetitions. This controlled setup allows us to isolate and quantify the effect of node location on visibility, independent of hardware or configuration artifacts, and provides a foundation for developing customized task allocation strategies.

**Figure 1:**
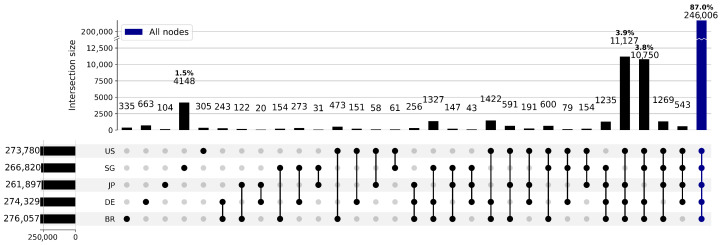
Heterogeneity of network visibility across nodes: IP address detection results. The significant non-overlapping areas visually confirm the systematic differences in node visibility. Only 87% of the target IPs were visible to all nodes; the remaining IPs exhibited node-specific visibility patterns. Intelligent systems must treat nodes as heterogeneous, not interchangeable.

**Figure 2:**
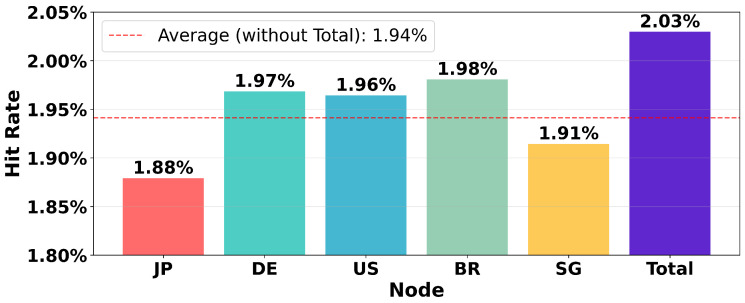
Hit rates by node and total. The average hit rate across all nodes is only 1.94%, indicating that active IPs in cyberspace are highly sparse. Leveraging this sparsity effectively could eliminate a substantial number of unnecessary probes.

The results are presented in Figure 1 and Figure 2. Figure 1 reveals significant and systematic disparities in the results of the investigation of the same targets between nodes located in different regions, indicating that each node has a unique and partial view of the Internet. On average, a single node can only detect about 95% of the total assets, with the node JP having the lowest coverage at 92%. Only 87% of the target IPs were visible to all nodes. The remaining 13% exhibited distinct node-specific visibility patterns, with approximately 2% of the assets accessible only from a single probing source. This implies that these IPs can only be discovered if assigned to that specific node. Although this proportion may appear small, in absolute terms, it translates to 36,875 IPs that could be missed due to inappropriate node selection - and this is only within the 1800 CIDR blocks sampled. Given that the full MaxMind database contains 629,757 CIDR blocks, the number of potentially overlooked assets in an Internet-wide scan cannot be overlooked. If such visibility heterogeneity is ignored during distributed scanning and tasks are assigned indiscriminately, the impact on coverage could be substantial.

Moreover, the remarkably low average hit rate of only 1.94% shown in Figure 2 indicates that a significant portion of the blocks and IPs in the IPv4 address space remain inactive, and the active IPs are highly sparse. If we can take advantage of this sparsity and detect these inactive blocks in advance, the probing overhead could be significantly reduced.

### 2.2. Limitations of Existing Allocation Strategies

Despite the critical importance of accounting for visibility heterogeneity in distributed probing, existing task allocation strategies often fail to adequately leverage these differences, which may cause insufficient coverage, redundant probing, and ineffective measurements.

**Random allocation:** The most common baseline; it treats all nodes as equal and distributes targets randomly. This approach does not attempt to match targets with nodes that can see them, leading to suboptimal coverage and inefficient use of resources, as demonstrated in our evaluation (Section 4). Previous studies have proposed various distributed scanning frameworks [29,30,31]. These systems focus primarily on achieving load balance, improving overall efficiency, and improving the flexibility and scalability of the system. However, they share a common limitation: treating both nodes and subtasks as homogeneous and interchangeable. Their task allocation strategies are typically randomized, involving either a central controller assigning tasks uniformly or nodes automatically claiming subtasks based on their current load.

**Geographic heuristics:** The paper [32] showed that the regional characteristics of scanners greatly affect the scan rate and the port connection latency within the same country or region is lower than between countries and regions. Therefore, they cluster scanners based on geographical location and further assign tasks regionally using historical average latency tables, thereby reducing average detection delay and improving task completion efficiency. However, the resources of the probing node are limited; we cannot always rely on nodes located within the target region. Assigning subtasks based solely on geographic clusters also overlooks the heterogeneity of IP responsiveness within regions. Additionally, constructing an average latency table requires historical full-coverage probing data, which incurs significant computational and management overhead. However, the resources of probing nodes are limited, and we cannot always guarantee the availability of nodes within the target region. Assigning sub-tasks based solely on geographical distribution also overlooks the heterogeneity of IP responsiveness within the same region. Furthermore, constructing an average latency table relies on historical full-coverage probing data, which entails significant computational and management overhead.

Therefore, it is imperative to develop a lighter method for evaluating distributed node capabilities and an intelligent task allocation strategy that effectively leverages limited probing resources to maximize coverage and accuracy.

### 2.3. Problem Formalization and Key Insight

The Distributed Scanning Task Allocation Problem can thus be formalized as follows. Given a set of targets *C* (e.g., a list of IP addresses or CIDR blocks) and a set of distributed nodes *N*, find an assignment A:C→N that maximizes the number of responsive targets found, minimizes the total completion time, and balances the workload across *N*, subject to the constraint that a node can only probe the targets it can reach. Therefore, a pivotal and urgent task is to define the theoretical foundation for intelligent task allocation.

The key principle is to assign tasks based on node specialties to ensure workload equilibrium, and the primary challenge is to quickly and efficiently quantify and evaluate the coverage capacity of each node. Our proposed system, VistaScan, solves this problem by the key insight that while nodes exhibit vastly different visibility globally, the visibility pattern within a specific CIDR block remains highly consistent from any single node’s perspective. We formalize this property as Visibility Consistency and formally validate it in Appendix C.

**Definition** **1** (Visibility Consistency)**.** *For a given CIDR block $B=\{ip_{1},ip_{2},\ldots ,ip_{m}\}$ and a set of distributed probing nodes $N=\{n_{1},n_{2},\ldots ,n_{k}\}$, we define* visibility vector *for an IP address $ip_{i}$ as a binary vector $\vec{v_{i}}=\left(V\left(ip_{i},n_{1}\right),V\left(ip_{i},n_{2}\right),\ldots ,V\left(ip_{i},n_{k}\right)\right)$, where $V(ip_{i},n_{j})$ indicates the visibility of $ip_{i}$ from node $n_{j}$. The degree of* Visibility Consistency *within block B is then quantified as the average pairwise Jaccard similarity between the visibility vectors of all IP pairs in B:*

$$\mathrm{Consistency}\left(B\right)=\frac{2}{m(m-1)}\sum_{i=1}^{m-1}\sum_{j=i+1}^{m}J\left(\vec{v_{i}},\vec{v_{j}}\right)$$

*where $J(\vec{v_{i}},\vec{v_{j}})$ is the Jaccard similarity coefficient:*

$$J\left(\vec{v_{i}},\vec{v_{j}}\right)=\frac{|\vec{v_{i}}\cap \vec{v_{j}}|}{|\vec{v_{i}}\cup \vec{v_{j}}|}=\frac{\sum_{k=1}^{k}\min\left(v_{i,k},v_{j,k}\right)}{\sum_{k=1}^{k}\max\left(v_{i,k},v_{j,k}\right)}$$

**Figure 3:**
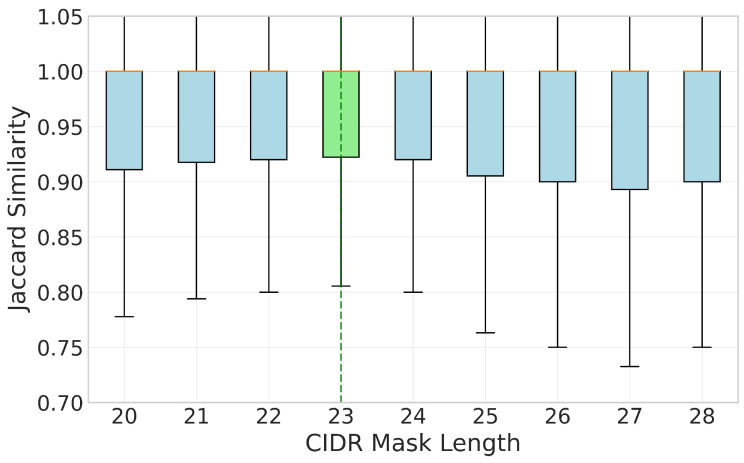
Distribution of Jaccard similarity across different CIDR sizes. The /23 block size exhibits a higher lower bound.

A higher consistency score indicates that the visibility patterns of IPs within the block are more homogeneous, justifying the use of representative anchor IPs for the entire block. We also quantify the Visibility Consistency of various sizes of CIDR blocks and one of the figures is shown as Figure 3, indicating that /23 is the optimal block size for overall consistency (see Appendix C.3 for details).

## 3. VistaScan System Design

The core idea of the *VistaScan* system is to assign the probing tasks of different network blocks to the best-suited probing nodes. Based on the principle of *Visibility Consistency*, the visibility of representative *anchor IPs* by a node can characterize its visibility of the entire block. This allows for the assessment of a node’s capability without the need for a full-block exhaustive probe.

**Figure 4:**
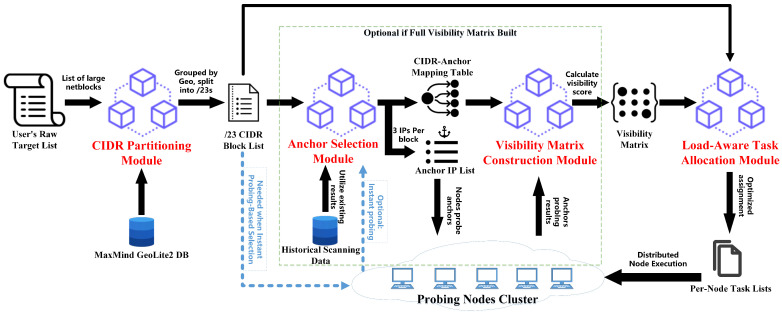
VistaScan system architecture overview. The system consists of four main modules: CIDR partitioning, anchor selection, visibility matrix construction, and load-aware task allocation.

The architecture of VistaScan, shown in Figure 4, is structured as a modular orchestration designed to transform a raw set of scanning targets into an optimally distributed task assignment. The system begins with *CIDR Partitioning Module*, which ingests user-defined targets and leverages the MaxMind GeoLite2 database to group them by autonomous system or geography before systematically splitting them into consistent /23 CIDR blocks. These standardized blocks then pass to the *Anchor Selection Module*, which identifies a small set of representative IP addresses (anchors) for each block in two optional ways. Subsequently, the *Visibility Matrix Construction Module* coordinates a lightweight validation probe where all distributed nodes probe these anchors, resulting in a visibility matrix that quantifies the coverage effectiveness of each node for each block. Finally, the *Load-Aware Task Allocation Module* consumes this matrix to intelligently assign each CIDR block to the most capable node while maintaining global load balance across the entire system. If a network-wide visibility matrix is already available, the two modules within the dashed box can be skipped, and the partitioned user tasks can be directly allocated based on this pre-built matrix, thereby further reducing system overhead. This orchestrated workflow ensures that the expansive scanning task is decomposed and allocated in a manner that maximizes coverage and efficiency by explicitly accounting for the inherent network visibility constraints of each probing node in a distributed probing scenario.

### 3.1. CIDR Partitioning

The CIDR Partitioning Module preprocesses the user’s raw input targets, which may be a list of individual IP addresses or large netblocks (e.g., a /12), and transforms them into a canonical set of mainly /23 CIDR blocks optimized for high Visibility Consistency, forming the foundational unit for subsequent processing of anchor selection and task allocation. This module optimizes load balance by refining subtasks into finer-grained units and operates in two stages.

1. **IP-to-Country Mapping:** Each IP address is mapped to its corresponding country or region using the MaxMind GeoLite2 database. This initial grouping takes advantage of the premise that IPs within the same geographic region are more likely to share network infrastructure and visibility characteristics.
2. **Canonical Splitting:** Address blocks larger than a /23 prefix are split into /23 CIDR blocks. This specific size was selected based on the analysis in Appendix C.3, which found it to be the optimal trade-off between Visibility Consistency and management overhead.

### 3.2. Anchor Selection

The target IP space is divided into no more than /23 blocks. For each block, we select a small set of anchor IPs to characterize its visibility. In our experiments, using three anchor IPs from different probing sources per block proved to be a practical and effective heuristic, balancing representativeness, robustness, and probing overhead. The theoretical criteria for anchor selection are detailed in Appendix D, and are supported by prior work that leverages limited sampling to infer broader network characteristics [7,12,28,33,34,35,36,37,38]. The optimal number of anchors may vary depending on network conditions and consistency levels, and is a subject for future parameter tuning. The selection of representative anchor IPs can be accomplished through two primary methods:

1. **Historical Data-Based Selection:** This approach utilizes existing probing results, requiring no additional probing overhead beyond the main task. Although computationally efficient, its main limitation is the potential lack of timeliness in anchor IP availability, as the historical data may not reflect the current network state.
2. **Instant Probing-Based Selection:** This method involves initiating a targeted, rapid random probe for a single IP within each target CIDR block by pre-configured nodes. The results of these probes are then aggregated to generate the final anchor list. The principal advantage of this method is the strong timeliness and freshness of the anchors obtained. However, it introduces additional probing overhead and latency before the main scan can begin.

For probing tasks with stringent timeliness requirements—such as post-incident assessments or scans in highly dynamic network environments—users can perform instant anchor scanning and visibility matrix construction on a per-task basis, accepting a modest increase in probing overhead in exchange for maximum freshness. This flexibility ensures that VistaScan can be tailored to a wide range of operational contexts.

Algorithm 1 demonstrates the efficient retrieval and anchor generation process based on historical data. Its core operation involves, for each block, performing an efficient lookup to find belonging IPs from each node’s perspective, randomly selecting one candidate IP per node, and finally deduplicating and sampling up to three unique IPs to serve as the final anchors for that block. The process outputs a comprehensive list of all representative IPs and a mapping of each CIDR block to its anchors. The instant probing method differs only in its data source, consuming a live stream of probe results from each node instead of querying static historical files.
**Algorithm 1** Historical Data-Based Anchor IP Selection**Require:** Set of distributed nodes: $N=\{n_{1},n_{2},\ldots ,n_{k}\}$**Require:** Sorted IP lists from each node’s scan results: $L_{n_{1}},L_{n_{2}},\ldots ,L_{n_{k}}$**Require:** List of target CIDR blocks: *C***Ensure:** Representative IP list *R***Ensure:** Mapping *M* from CIDR blocks to representative IPs   1: R←{}▹ Initialize representative IP list   2: M←{}▹ Initialize CIDR-to-IP mapping   3: **for each** CIDR block $c_{i}$ **in** *C* **do**   4:       S←{}▹ Initialize set for candidate IPs per block   5:       **for each** node $n_{j}$ **in** *N* **do**   6:             $I_{\mathrm{candidate}}\leftarrow \mathrm{FindIPsInCIDR}\left(L_{n_{j}},c_{i}\right)$▹ Binary search for IPs in CIDR   7:             **if** $I_{\mathrm{candidate}}\neq \emptyset$ **then**   8:                 $ip_{\mathrm{selected}}\leftarrow \mathrm{RandomChoice}\left(I_{\mathrm{candidate}}\right)$▹ Random selection per node   9:                 $S\leftarrow S\cup \{ip_{\mathrm{selected}}\}$▹ Add to candidate set 10:             **end if** 11:       **end for** 12:       $S_{\mathrm{unique}}\leftarrow \mathrm{RemoveDuplicates}\left(S\right)$▹ Preserve order while removing duplicates 13:       **if** $S_{\mathrm{unique}}\neq \emptyset$ **then** 14:             $F\leftarrow \mathrm{RandomSample}(S_{\mathrm{unique}},\min(3,|S_{\mathrm{unique}}\left|)\right)$▹ Select up to 3 unique IPs 15:             R←R∪F▹ Add to final representative list 16:             **for each** IP $ip_{k}$ **in** *F* **do** 17:                   $M\leftarrow M\cup \{\left(c_{i},ip_{k}\right)\}$▹ Add to CIDR-to-IP mapping 18:             **end for** 19:       **end if** 20: **end for** 21: **return** R,M

This algorithm takes sorted IP lists from historical scanning data from different nodes and a list of target CIDR blocks as input. Its design directly addresses the core requirements of representativeness and diversity by leveraging a key insight: the collective historical data from all nodes provide the most unbiased view of a block’s visibility landscape. By performing an efficient lookup into each node’s sorted IP list, it ensures computational scalability. The strategy of randomly selecting one candidate IP from each node’s perspective inherently incorporates the necessary discriminative power and mitigates single-point visibility bias, as the final anchor set is distilled from a pool that reflects the heterogeneous nature of the network. Finally, the deduplication and sampling step robustly handles real-world data imperfections, enforcing a multi-anchor strategy to balance robustness with low overhead. This synergy of efficiency, built-in diversity, and practical robustness makes the algorithm a critical component for building the foundational data structures of our distributed scanning system.

### 3.3. Visibility Matrix Construction

The Visibility Matrix *M* is a core data structure that encodes the visibility score M[n,c] for each node–block pair (n,c), where n∈N is a node and c∈C is a CIDR block. The score M[n,c] is defined as the fraction of anchor IPs in block *c* that node *n* can successfully probe, formalized as:

$$M\left[n,c\right]=\frac{\left|\{a\in \mathrm{Anchors}(c)\;|\;\mathrm{Probe}(n,a)=\mathrm{success}\}\right|}{\left|\mathrm{Anchors}(c)\right|}$$

where Anchors(c) is the set of anchor IPs for block *c* and Probe(n,a) denotes the result of node *n* probing anchor *a*.

Algorithm 2 outlines the construction process. It first precomputes a reverse lookup table IP2Block to efficiently map any anchor IP back to its parent CIDR block (lines 1–3). The reverse mapping IP-to-Block ensures that determining the parent block of any visible IP is an O(1) operation, which is crucial given the scale of Internet-wide scanning. For each node *n*, it processes the set of anchor IPs visible to *n* ($V_{n}$). For each visible IP, it uses the IP2Block map to increment a counter for the corresponding block (lines 5–9). Finally, the visibility score for each block is computed by normalizing the count of visible anchors by the total number of anchors in that block (lines 10–13).
**Algorithm 2** Build Visibility Matrix *M***Require:** $\mathrm{Anchors}:C\to A^{*}$▹ Map: Block *c* to its anchor lists   1: $V:N\to 2^{A}$▹ Map: Node *n* to its set of visible anchors   2: N,C▹ Lists of all nodes and CIDR blocks**Ensure:** Visibility Matrix M[N,C]   3: IP2Block←{}▹ Initialize IP-to-Block mapping   4: **for** 
c∈C
**do**   5:         **for**  a∈Anchors(c) **do**   6:                IP2Block[a]←c   7:         **end for**   8: **end for**   9: **for** n∈N**do**▹ For each node 10:       Count←DefaultDict(int)▹ Initialize per-block counter 11:       **for**  a∈V(n) **do**▹ For each anchor visible to node *n* 12:              **if** a∈IP2Block **then** 13:                    c←IP2Block[a] 14:                    Count[c]←Count[c]+1 15:              **end if** 16:       **end for** 17:       **for**  c∈C **do**▹ Compute final scores 18:              M[n,c]←Count[c]/|Anchors(c)| 19:       **end for** 20: **end for**

This process efficiently transforms raw probe results into a quantitative suitability matrix, which is the final, critical input for the subsequent task allocation stage.

### 3.4. Load-Aware Task Allocation

The central component of VistaScan before probing is its intelligent task allocation algorithm, which optimally assigns CIDR blocks to distributed nodes based on pre-computed visibility scores while maintaining load balance. The algorithm maximizes coverage probability by leveraging the Visibility Consistency property and ensures efficient resource utilization through dynamic load-aware scheduling.

Algorithm 3 encapsulates the main allocation logic. Process each CIDR block c∈C by first checking if any node has positive visibility (M[n,c]>0). For such blocks, it identifies the node(s) with the highest visibility score M[n,c]. If multiple nodes achieve the maximum score, the algorithm selects the currently least-loaded node $n^{*}$ to maintain the load balance. The selected node is assigned to the block *c* and its load count increases.

The algorithm separates blocks into two queues based on the availability of visibility information. The priority queue ($C_{\mathrm{priority}}$) contains blocks with established visibility patterns, which are assigned to nodes that have demonstrated the highest historical visibility scores. Tie-breaking based on current load ensures balanced distribution, which prevents node overload and ensures efficient resource utilization. Additionally, the algorithm maintains the linear time complexity O(|C|×|N|), guaranteeing scalability for large-scale Internet-wide scanning tasks. Our system and algorithm are fully scalable to a larger number of nodes.
**Algorithm 3** Load-Aware Task Allocation**Require:** C: List of CIDR blocks   1: M: Visibility matrix (N nodes × C blocks)   2: N: List of distributed nodes**Ensure:** A: Block-to-node assignment   3: L: Node load counts   4: A←{}, $L_{n}\leftarrow 0$ for all n∈N   5: $C_{\mathrm{priority}}\leftarrow \left[\right]$▹ Blocks with max(M[:,c])>0   6: $C_{\mathrm{fallback}}\leftarrow \left[\right]$▹ Blocks not in or with max(M[:,c])=0   7: **for each** block c∈C **do**   8:       **if** max(M[:,c])>0 **then**   9:             $C_{\mathrm{priority}}.\mathrm{append}\left(c\right)$ 10:       **else** 11:             $C_{\mathrm{fallback}}.\mathrm{append}\left(c\right)$▹ For future implementation 12:       **end if** 13: **end for** 14: **for each** block $c\in C_{\mathrm{priority}}$ **do** 15:       $s_{\max}\leftarrow \max\left(M\left[:,c\right]\right)$ 16:       $N_{\mathrm{candidate}}\leftarrow \left\{n\in N\;|\;M\left[n,c\right]=s_{\max}\right\}$ 17:       **if** $|N_{\mathrm{candidate}}|=1$ **then** 18:             $n^{*}\leftarrow \mathrm{the}\;\sin\mathrm{gle}\;\mathrm{node}\;\mathrm{in}\;N_{\mathrm{candidate}}$ 19:       **else** 20:             $n^{*}\leftarrow \underset{n\in N_{\mathrm{candidate}}}{\mathrm{argmin}}\;L_{n}$ 21:       **end if** 22:       $A\left[c\right]\leftarrow n^{*}$, $L_{n^{*}}\leftarrow L_{n^{*}}+1$ 23: **end for** 24: **return** A, L

Currently, we only perform the priority allocation for CIDR blocks that are present in the visibility matrix. Those blocks not included in the matrix indicate that no node currently has visibility in them and are therefore discarded directly, a decision grounded in our nodes’ capability and the sparsity of active IPs (as shown in Figure 2). The fallback queue ($C_{\mathrm{fallback}}$) is reserved for blocks lacking visibility data, providing a framework for future work on complete coverage strategies.

## 4. Evaluation

We present a comprehensive quantitative evaluation of VistaScan against multiple baselines in Global, Regional, and Special-Block tasks. Section 4.1 covers the experimental environment. Section 4.2, Section 4.3 and Section 4.4 demonstrate the performance of VistaScan.

### 4.1. Experimental Setup

To thoroughly evaluate the performance of VistaScan, we conducted extensive experiments in a variety of scenarios. Our testbed, target selection, and evaluation methodology were designed to ensure a fair and comprehensive comparison against established baselines.

**Testbed.** Our distributed probing infrastructure consisted of five nodes strategically deployed throughout the world using commercial cloud services. The details of each node are summarized in Table 1. All nodes were equipped with identical hardware specifications and a 100 Mbps network interface to eliminate performance disparity caused by resource differences. This homogeneous configuration allows us to isolate and attribute any performance variations solely to our task allocation strategy and network visibility, rather than to raw computational or network power.

**Scanning Rate.** To ensure fairness and prevent measurement artifacts caused by local congestion or packet loss, all experiments used a fixed scanning rate of **1500 pps** per node. This value was determined to be the optimal global rate by the calibration process described in Appendix B.

**Scanning Tasks and Targets.** Table 2 summarizes the three scanning tasks designed to thoroughly evaluate VistaScan under different conditions. The Global task consisting of 3 countries from each of 6 continents was designed to assess worldwide coverage. The Regional task focused on the regions where our nodes are located. The targets of these two tasks were randomly selected from the MaxMind GeoLite2 database to ensure unbiased and generalizable results. The Special-Block task was designed to stress-test the system’s ability to handle challenging blocks where node visibility is highly heterogeneous, in which the targets were manually selected based on our scanning experience.

**Baseline Comparisons.** We implemented five baseline methods for a comprehensive and rigorous comparison. This design allows us to dissect and isolate the contribution of each individual component within VistaScan.

- **Centralized:** A single node (e.g., US) scans the entire target list. This baseline highlights the advantages of a distributed architecture over a traditional centralized approach.
- **Union-of-All:** All five nodes independently and simultaneously scan the *entire* target set. The final result is the Union-of-All their outputs. This provides the **absolute upper bound** for the coverage achievable by any distributed system with these five vantage points, serving as a key reference for evaluating the coverage *efficiency* of VistaScan and other methods.
- **Naive-Distributed-Random:** The original, unsplit target list of IPs is randomly and uniformly assigned to the five nodes. This evaluates a simple distributed strategy without intelligent partitioning or allocation.
- **Partitioned-Distributed-Random:** The target IP space is first partitioned into /23 CIDR blocks using the VistaScan CIDR partitioning module. Then, this list of blocks is randomly and uniformly assigned to the five nodes. This baseline isolates the performance gain that is only attributable to the CIDR partitioning step.
- **Geo-Heuristic:** Targets are assigned to the geographically closest node to them (for example, Brazilian IPs to the BR node). This baseline is only used for the *Regional task* and represents a common geographic-driven heuristic.

**Evaluation Metrics.** We evaluated the performance of each method based on the following metrics:

- **Global Coverage:** The total number of unique IP addresses that respond. To contextualize this absolute value and measure the efficiency of a method’s coverage, we also report it as a percentage of the total unique IPs found by the *Union-of-All* baseline. This relative measure indicates how close a method gets to the theoretical maximum coverage achievable with the given set of vantage points.
- **Total Packets Sent:** The aggregate number of probe packets sent by all nodes, indicating the overall network overhead and efficiency of the method.
- **Total Completion Time:** The wall-clock time from the start of the first probe to the completion of the last probe across all nodes.
- **Node Load Balance:** The standard deviation of the number of scanning IP addresses assigned to each node, quantifying the fairness workload distribution.

### 4.2. Coverage Analysis

The paramount goal of a distributed scanning system is to maximize coverage. Our evaluation demonstrates that VistaScan consistently achieves near-optimal coverage across all scenarios, a direct result of its core design principle: Intelligently assigning each CIDR block to the node with the highest probability of seeing it.

As synthesized in Table 3, VistaScan’s coverage remains exceptionally high and stable, reaching 97.95%, 99.05%, and 97.58% in the Global, Regional, and Special-Block tasks, respectively. This performance places it within 2-3% of the theoretical maximum (Union-of-All) and significantly outperforms all centralized and naive distributed baselines. Notably, in the Regional task, VistaScan matches the performance of Geo-Heuristic (99.05% vs. 99.35%) with significantly lower scanning overhead time consumption and without relying on external geographical databases, demonstrating the sufficiency of its empirically measured visibility matrix.

The Special-Block task serves as a stress test. These blocks exhibit poor Visibility Consistency, comprising targets with pronounced visibility heterogeneity. VistaScan’s robust performance here (97.58%) underscores its effectiveness even in the most challenging network environments. Crucially, this high coverage is not achieved by brute force, but through precision: the system allocates tasks based on the visibility matrix, ensuring each block is probed by its most capable node. Our method can also effectively avoid assigning tasks to nodes with poor coverage capabilities for specific blocks, such as the JP node.

**Figure 5:**
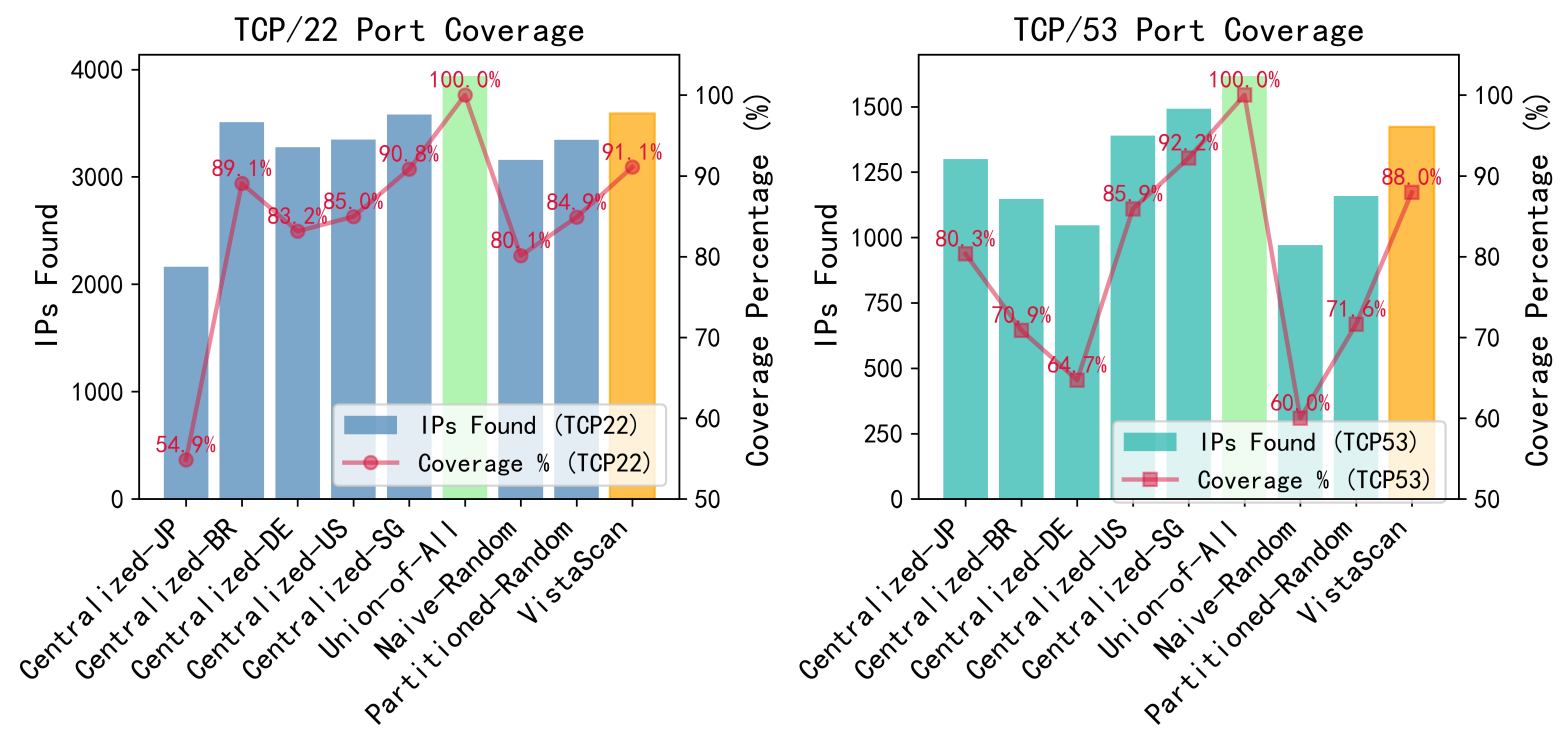
Cross-port coverage performance: Applying TCP/80 Visibility Matrix to TCP/22 and TCP/53 Scanning. VistaScan maintained high coverage of 91.09% and 87.95%.

VistaScan also features Cross-Port Applicability. As shown in Figure 5, we found that the visibility matrix constructed from one TCP port (TCP/80) is applicable to other TCP ports. When applied to scanning TCP/22 and TCP/53 ports, VistaScan maintained a high coverage of 91.09% and 87.95%, respectively. Within the scope of the TCP protocol that we probed, this cross-port applicability indicates that visibility patterns exhibit cross-port generalization capabilities. It enhances the practicality of VistaScan, as a single profiling phase can optimize subsequent scans for multiple TCP services, dramatically reducing operational overhead.

### 4.3. Efficiency Analysis: Overhead and Completion Time

**Figure 6:**
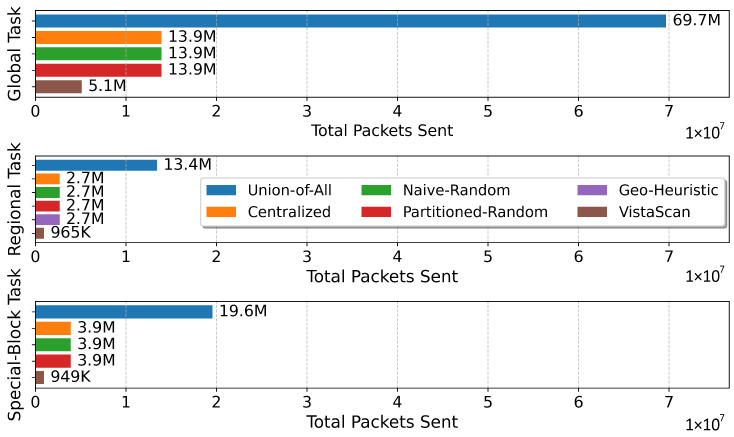
Total packets sent.

**Figure 7:**
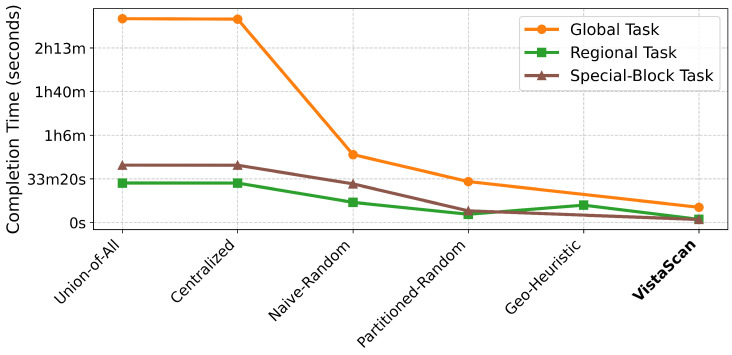
Total completion time.

Beyond coverage, VistaScan’s paramount advantage is its revolutionary efficiency, simultaneously minimizing two critical resources: network overhead and completion time. As shown in Figure 6 and Figure 7, VistaScan consistently demonstrates orders-of-magnitude improvements in efficiency.

**Network Overhead:** As illustrated in Figure 6, VistaScan drastically reduces the total number of packets sent. Compared to the baseline methods that scan all targets (approx. 2.7 M–39.2 M packets, depending on the task), VistaScan reduces the network overhead by 64% to 93%. This reduction is the direct consequence of its decisive filtering strategy: any CIDR block with no visible anchor IPs in the visibility matrix is pruned from the task list entirely. This avoids futile probing of network blind spots, constituting a fundamental shift from exhaustive scanning to targeted, intelligent probing.

**Completion Time:** Figure 7 shows the total completion time. VistaScan completes scans 13 to 19 times faster than centralized baselines. This speedup is a compound effect of (1) reduced total workload (fewer packets to send) and (2) highly efficient parallelization due to excellent load balance (analyzed in Section 4.4). For example, in the Global task, VistaScan was completed in 11 min and 34 s, compared to over 2.5 h for the centralized approaches. This efficiency enables near-real-time Internet-scale scanning, opening new possibilities for frequent monitoring and rapid incident response.

**Cost of Pruning:** While pruning blocks with no visible anchors drastically reduces probe volume, it inevitably forgoes scanning some IPs. To assess the potential loss of coverage, we examined the pruned blocks in our Global task. Among these blocks, the proportion of responsive IPs was extremely low—less than 0.005% of the IPs in these blocks responded to the probes, and the vast majority of pruned blocks contained no active hosts at all. In practice, VistaScan incurs only about a 2.0% coverage loss compared to the Union-of-All baseline (which represents the theoretical maximum achievable with our five vantage points). Importantly, this loss already includes the inherent coverage deficit of any single node scan (as seen in the Centralized baselines). Therefore, the additional coverage loss attributed to pruning is negligible while yielding substantial savings in probing overhead.

### 4.4. Load Balance Analysis

The efficiency of a distributed system depends on how well it balances workload across its nodes. Poor load balance leads to resource idle time and becomes the bottleneck for completion time.

**Figure 8:**
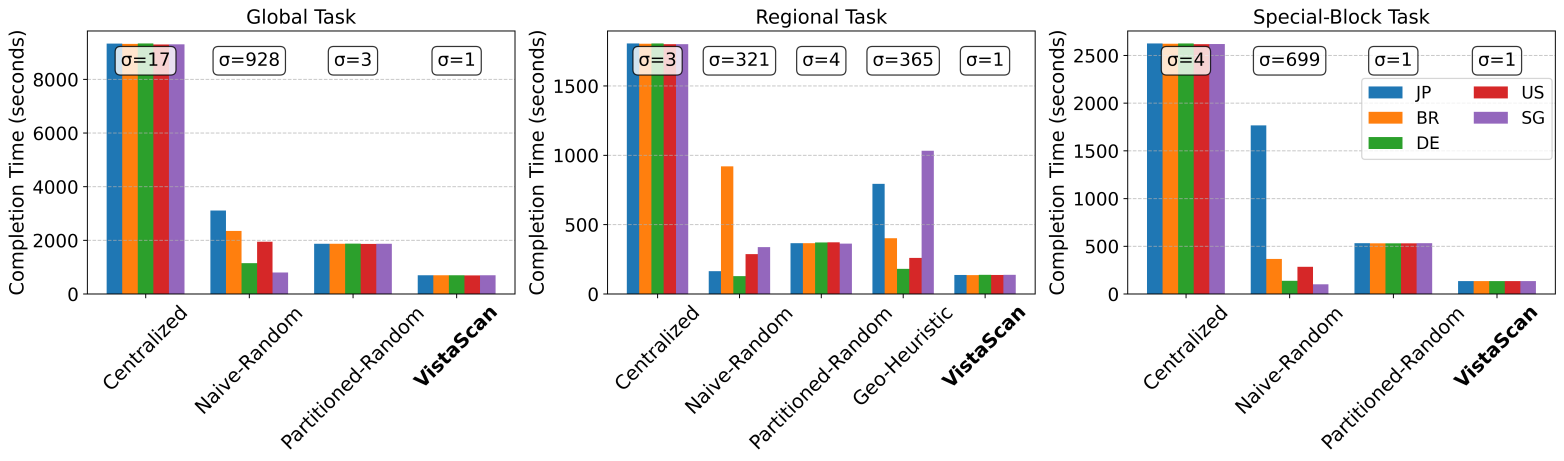
Load balancing performance across three probing scenarios. VistaScan demonstrates consistently excellent load balance across all nodes and tasks, while other distributed methods (Naive-Random, Geo-Heuristic) suffer from significant imbalance, leading to longer overall completion times.

Our analysis reveals that VistaScan’s allocation algorithm achieves near-perfect load balance. As depicted in Figure 8, task completion times between all five nodes are virtually identical in all VistaScan scenarios. This is because its allocation algorithm is not only visibility-aware, but also load-aware; when multiple nodes have similarly high visibility scores for a block, the algorithm assigns it to the currently least-loaded node.

In stark contrast, other distributed strategies show a severe imbalance. The Naive-Random method leads to a highly skewed distribution of work, with the slowest node taking up to 3.8 times longer than the fastest to finish its assigned segment. The Geo-Heuristic method also exhibits a significant imbalance in the Regional task, as geographical proximity does not perfectly correlate with scan workload or link quality, nor does it guaranty balanced task allocation across regions. Compared to *Naive-Distributed-Random*, the *Partitioned-Distributed-Random* method equipped with the CIDR Partitioning Module achieves a more uniform distribution of tasks, significantly reduces the overall completion time, and exhibits superior load balance.

## 5. Discussion and Limitations

Our experimental results demonstrate that VistaScan achieves significant performance improvements in various scanning scenarios. In the global scenario, VistaScan achieves higher coverage than all other methods while requiring only 36.7% of packets and 7.4% of the time compared to single-point centralized robing. In regional scanning, VistaScan achieves a nearly identical hit rate to Geo-Heuristic while using only 37% of its probe packets and 17.4% of its time, along with a more balanced node load. In addition, our method excels particularly at Special-Block tasks, outperforming all other approaches across all metrics and approaching the theoretical maximum coverage. The success of our system hinges on two validated core principles: the Visibility Consistency phenomenon within CIDR blocks and the systematic heterogeneity of visibility across geographically distributed nodes. By quantifying the latter into a Visibility Matrix and leveraging the former to make this matrix efficient to construct, VistaScan enables an intelligent task allocation strategy that is both precise and practical, moving the field from ad hoc and static allocation strategies to a data-driven and adaptive framework, fundamentally improving the efficiency and effectiveness of distributed Internet measurement. VistaScan’s advantages can be summarized as follows:

- **Streamlining Evaluation:** The Visibility Consistency principle enables the inference of full-block visibility from a few anchor IPs, dramatically lowering the assessment overhead through targeted probing rather than full-block scanning.
- **Quantifying Capability:** We design two anchor selection methods and then build the Visibility Matrix through lightweight probing of anchor IPs, in which are the per-node visibility scores for each block.
- **Optimizing Assignment:** Using the Visibility Matrix to assign each block to the node with the highest probability of seeing it (the “most capable” node), while simultaneously balancing the load.
- **Pruning Infeasible Work:** Intelligently discarding blocks for which no node has significant visibility, thus eliminating ineffective probing.

However, we acknowledge several limitations of our current work that point to future research directions.

**Anchor Freshness and Matrix Validity:** A fundamental assumption of VistaScan is that the visibility matrix provides a reasonably stable representation of the network state. Although network paths are generally stable, events such as routing changes, outages, or filtering updates could stale the matrix over time, leading to task misassignment or erroneous block pruning. To address this, a production system requires lightweight mechanisms for periodic or triggered re-validation—dynamically adjusting probing frequency based on observed network dynamics (e.g., failure rates). Our future work will systematically address temporal robustness along two directions: we plan to evaluate the effective duration of a single constructed visibility matrix over longer time scales, and explore two lightweight update mechanisms—periodic active probing of anchor IPs (e.g., weekly), and online learning that refines visibility scores using incidental observations from main scans. Together, these approaches will enable VistaScan to adapt to gradual network changes while preserving efficiency, ensuring its viability for sustained real-world deployment.

**Coverage–Completeness Trade-off:** The strategy of pruning blocks with no visible anchors is the primary driver of efficiency gains. However, it inherently sacrifices absolute completeness (as achieved by the Union-of-All baseline) for efficiency. This is a deliberate and beneficial engineering trade-off for most scanning use cases. Nevertheless, for applications where finding every single responsive host is critical, our method could be extended to include a fallback mechanism where pruned blocks are periodically scanned by a random node or all nodes at a very low rate to check for changes. This is why we maintain the fallback queue ($C_{\mathrm{fallback}}$) mentioned in Section 3.4. Subsequently, a low-frequency scanning and update mechanism will be established for it.

**Anchor Count Selection and Extension to UDP Scanning:** While our empirical results demonstrate the effectiveness of using three anchors per block (each from a diverse source), the optimal number warrants further investigation, as it may depend on factors such as block size, network stability, and the trade-off between accuracy and overhead. Future work could explore adaptive anchor selection mechanisms that dynamically adjust the anchor count based on intra-block consistency estimates or historical data. In addition, UDP’s connectionless nature, reliance on ICMP, and distinct firewall handling may produce visibility patterns that differ from TCP. While VistaScan currently focuses on TCP, future work should explore its extension to UDP. This requires addressing challenges in probing strategy, response ambiguity, and timeout handling, as well as validating whether Visibility Consistency holds for UDP services. Such research would broaden the applicability of visibility-aware distributed scanning.

**Handling of Ultra-Low Consistency Blocks:** No real-world system can rely on perfect statistical regularity, and our design pragmatically exploits the strong statistical trend of Visibility Consistency while handling outliers gracefully. We acknowledge that a small subset of blocks exhibit very low intra-block consistency, typically due to complex network configurations such as multihoming or fine-grained, IP-specific filtering policies. For such blocks, a single optimal node may not provide adequate coverage, as visibility can vary significantly even within a /23 prefix. Two promising directions for future work can address this limitation: (1) further subdividing these blocks into smaller granularities (e.g., /24 or /25) to restore high consistency with minimal overhead; and (2) employing multi-node coordinated probing, where tasks are distributed among a set of complementary nodes to maximize coverage. We plan to explore these optimizations in future work.

## 6. Related Works

Our work is based on and intersects with several key areas of Internet measurement and distributed systems research.

**Centralized High-Speed Scanning**. Modern scanning tools such as Nmap [24], ZMap [39], Masscan [23], Zippier ZMap [40], ZGrab2 [41], and LZR [42] revolutionized Internet-wide scanning by demonstrating that the entire IPv4 address space can be scanned in minutes from a single machine. With the support of high-performance I/O frameworks such as PF-RING [43], Packetshader [44] and netmap [45], port scanning time can be significantly reduced. VistaScan does not seek to replace these tools, but rather to extend their centralized paradigm. We address their inherent limitations: single-point visibility blind spots, susceptibility to IP blocking, and the physical bottleneck of a single source. VistaScan adopts their high-performance probing engines, but distributes the effort intelligently across nodes.

**Distributed Measurement Systems**. Distributed scanning technology reduces the time required for large-scale scanning and detection by decomposing scanning tasks and distributing them across multiple nodes. The research community has developed several distributed scanning systems [29,30,31,32]. However, their native task allocation mechanisms are often simplistic, relying on random assignment or geographic heuristics. VistaScan contributes a novel, automated, and intelligent allocation layer that could be deployed on such platforms to dramatically increase the efficiency and coverage of experiments run on them.

**Efficient Predictive Scanning Systems**. Predictive scanning offers a novel approach to improving the efficiency of large-scale scanning operations. In contrast to distributed scanning systems that minimize scanning duration through probing resource expansion and task partitioning, predictive scanning operates by narrowing the target space via a pregenerated prediction list. Previous works such as Smart Internet Probing [46] by training a unique XGBoost classifier [47], GPS [12], PMap [48], and IPREDS [10] have attempted to develop efficient predictive scanning systems capable of forecasting port activity on target hosts, thereby reducing the time required to discover a sufficient number of valid targets. However, after generating the prediction list, they still face the problem of determining which specific node(s) should execute the actual scanning tasks. VistaScan can act as the core distributed probing engine of these predictive systems, increasing the accuracy of their predictions effectively. Our system integrates the strengths of both distributed and predictive scanning, which not only increases available probing resources but also reduces the probing space, while intelligently assigning tasks to the most suitable nodes.

**Studies of Network Visibility**. Previous work has documented and analyzed the heterogeneity of views from different organizations [26,27], different scanning origins [28], different probing tools [40] and different probes [3], and the paper [25] further showed that web-based geoblocking is increasingly common. We have taken a step further by systematically investigating the extent to which the location of probing nodes influences the results of distributed measurements. In addition, VistaScan goes beyond observation to application. We are the first to formalize the “Visibility Consistency” hypothesis and use it as the foundational principle for building a practical, optimized distributed scanning system. Our work shows that these observed biases are not just noise to be accounted for, but a signal that can be exploited for significant performance gains.

In summary, VistaScan distinguishes itself by introducing a formal model of Visibility Consistency, which enables a lightweight node’s capabilities evaluation methods and a novel task allocation algorithm that are both more efficient and provide higher coverage than existing distributed approaches, while overcoming the visibility and efficiency limitations of centralized scanners.

## 7. Conclusions

In this paper, we introduced VistaScan, a novel system for optimizing distributed Internet-wide scanning based on the key insight of Visibility Consistency, that the visibility pattern among IP addresses is highly consistent within CIDR blocks. This principle enabled the construction of a lightweight Visibility Matrix via anchor-based probing and informed the design of a load-aware task allocation algorithm. Our comprehensive evaluation across Global, Regional, and challenging Special-Block tasks demonstrates that VistaScan consistently outperforms existing methods, achieving superior coverage by matching blocks to the most capable nodes while significantly improving efficiency through the pruning of unreachable targets and intelligent load balancing. In particular, the visibility matrix derived from one TCP port (TCP/80) proved highly effective for scanning other TCP ports (TCP/22, TCP/53), preliminarily underscoring the generalizability of our approach. VistaScan shows how a deeper understanding of network structure can lead to substantial practical gains, opening new avenues for building efficient, large-scale distributed measurement systems that are fine-grained, low-impact, and resource-efficient.

## Figures and Tables

**Table 1:** Configuration of distributed probing nodes.

Node	Location	OS	Hardware
JP	Tokyo, Japan	Ubuntu 20.04 LTS	2 vCPU/4 GB RAM/100 Mbps
BR	São Paulo, Brazil	Ubuntu 20.04 LTS	2 vCPU/4 GB RAM/100 Mbps
DE	Frankfurt, Germany	Ubuntu 20.04 LTS	2 vCPU/4 GB RAM/100 Mbps
US	San Jose, CA, USA	Ubuntu 20.04 LTS	2 vCPU/4 GB RAM/100 Mbps
SG	Singapore	Ubuntu 20.04 LTS	2 vCPU/4 GB RAM/100 Mbps

**Table 2:** Scanning tasks and targets.

Task Type	Countries	CIDR Blocks	Total IPs	Target Port(s)
Global	18	1800	13,936,863	TCP/80
Regional	5	500	2,689,122	TCP/80
Special-Block	15	53	3,915,152	TCP/80, 22, 53

**Table 3:** Synthesis of coverage results across all tasks.

Task	VistaScan	Union-of- All	Centralized					Naive- Random	Partitioned- Random	Geo- Heuristic
			JP	BR	DE	US	SG			
Global	97.95%	100.0%	92.35%	96.60%	96.08%	96.28%	96.08%	95.31%	96.08%	-
Regional	99.05%	100.0%	96.30%	96.52%	96.38%	98.94%	96.09%	96.36%	96.76%	99.35%
Special-Block	97.58%	100.0%	61.88%	93.52%	91.00%	90.03%	92.73%	66.23%	83.27%	-

**Figure A1:**
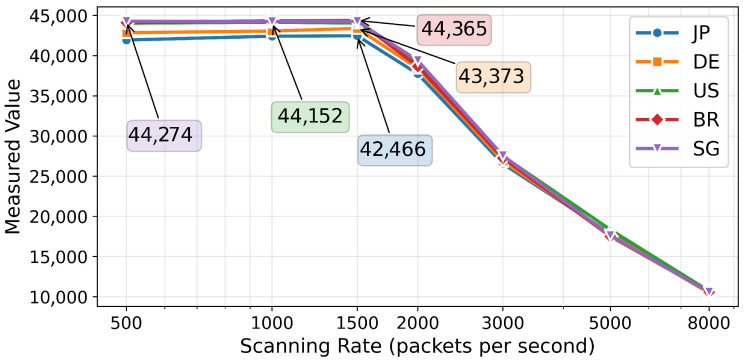
Rate vs. hit quantity for different nodes. Nodes BR, DE and JP peaked at 1500 pps. US and SG peaked at 1000 and 500 pps. All nodes’ performance sharply degrades beyond 1500 pps. Here, 1500 pps represents the optimal trade-off between efficiency and hit rate.

**Figure A2:**
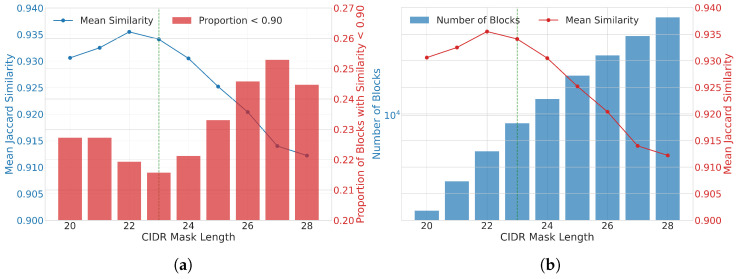
Characterization of Visibility Consistency across CIDR block sizes. (**a**) Trends of overall Consistency and low-Consistency proportions. (**b**) Trade-off: Number of blocks vs. Consistency.

## Data Availability

The data presented in this study are available upon request from the corresponding author.

## Appendix A. Ethical Considerations

We strictly adhered to responsible ethical and disclosure scanning practices. All probing was rate-limited to 1500 pps per node to avoid causing network congestion, performed without attempting exploitation, and targeted only at networks where such measurement is considered acceptable practice within the security research community. The goal of VistaScan is to improve the efficiency and accuracy of security and network research and to eliminate redundant scanning with the purpose of reducing the overall burden on Internet resources, not to facilitate malicious reconnaissance.

## Appendix B. Scanning Rate Calibration

In distributed measurement, the hardware performance of each node (e.g., CPU, network stack) can become a bottleneck for scanning rates. Excessively high packet sending rates may cause local packet loss, thereby distorting measurement results, while overly low rates under-utilize available resources. To ensure the reliability of all probing data, avoid distortion caused by a mismatch between hardware capability and scanning rate, and demonstrate that observed visibility differences are not due to misconfigured environments or suboptimal parameter settings, while also ensuring that all subsequent experiments operate at a sustainable and optimized rate, we first conducted a scanning rate calibration experiment for each node on 8 August 2025.

Each node was tasked with scanning a set of test targets at rates ranging from 500 to 8000 packets per second (pps), while the effective hit quantity (the number of successful responses) was measured. The test targets consisted of the top 500 CIDR blocks from the MaxMind GeoLite2 database. The scanning was performed using ZMap with TCP SYN scans targeting TCP/80. As shown in Figure A1, for our node configuration, the hit quantity of almost all nodes peaked and remained stable at 1500 pps. Beyond this rate, the hit rate declined significantly. Therefore, in all subsequent experiments, the scanning rate for each node was set uniformly to this calibrated and reliable optimal rate.

This calibration step ensures the rigor of our subsequent work in characterizing network visibility, confirming that the phenomena we observe are not mere noise. It also guarantees fairness in the comparative evaluation of method performance during experimentation, eliminating performance bias caused by improper node-specific configuration. All baseline methods are compared under optimal and consistent conditions.

## Appendix C. The Key Insight: Visibility Consistency Within CIDR Blocks

The heterogeneity in node-visibility shown in Section 2.1 reveals that the Internet is not a single uniformly visible space. The set of targets that a node can successfully probe is not random but is systematically influenced by its network location (Autonomous System), geographic region, and upstream providers. This leads to a fundamental question underpinning our work, which is that within a logically contiguous portion of the address space (a CIDR block), is the visibility of each IP consistent from the perspective of distributed vantage points? To answer this, we define Visibility Consistency.

### Appendix C.1. Problem Formulation and Definition

Let $B=\{ip_{1},ip_{2},\ldots ,ip_{m}\}$ be a CIDR block containing *m* IP addresses, and let $N=\{n_{1},n_{2},\ldots ,n_{k}\}$ be a set of *k* distributed probing nodes. The *visibility* of an IP address $ip_{i}$ from a node $n_{j}$ is defined as a binary function:

(A1) $$V\left(ip_{i},n_{j}\right)=\left\{\begin{array}{cc} 1 & \mathrm{if}\;ip_{i}\;\mathrm{is}\;\mathrm{reachable}\;\mathrm{from}\;(\mathrm{visible}\;\mathrm{to})\;n_{j}.\\ 0 & \mathrm{otherwise}. \end{array}\right.$$

For each IP address $ip_{i}\in B$, we define its *visibility vector* $\vec{v_{i}}$ as a *k*-dimensional binary vector that captures its global visibility pattern across all probing nodes:

(A2) $$\vec{v_{i}}=\left(V\left(ip_{i},n_{1}\right),V\left(ip_{i},n_{2}\right),\ldots ,V\left(ip_{i},n_{k}\right)\right)$$

The Visibility Consistency of block *B* is then quantified as the average pairwise Jaccard similarity between the visibility vectors of all IP pairs in *B*:

(A3) $$\mathrm{Consistency}\left(B\right)=\frac{2}{m(m-1)}\sum_{i=1}^{m-1}\sum_{j=i+1}^{m}J\left(\vec{v_{i}},\vec{v_{j}}\right)$$

where $J(\vec{v_{i}},\vec{v_{j}})$ is the Jaccard similarity coefficient between the vectors $\vec{v_{i}}$ and $\vec{v_{j}}$:

(A4) $$J\left(\vec{v_{i}},\vec{v_{j}}\right)=\frac{|\vec{v_{i}}\cap \vec{v_{j}}|}{|\vec{v_{i}}\cup \vec{v_{j}}|}=\frac{\sum_{l=1}^{k}\min\left(v_{i,l},v_{j,l}\right)}{\sum_{l=1}^{k}\max\left(v_{i,l},v_{j,l}\right)}$$

The value of Consistency(B) ranges from 0 to 1, where:

- Consistency(B)=1 indicates perfect consistency (all IPs in *B* have identical visibility patterns);
- Consistency(B)=0 indicates no consistency (all IP pairs have completely dissimilar visibility patterns);
- Higher values indicate stronger Visibility Consistency within the block.

In summary, Visibility Consistency refers to the phenomenon that within the same CIDR address block, the visibility patterns of different IP addresses, as observed by distributed probing nodes, are highly similar or consistent. In other words, if a node in a distributed network can successfully probe one IP within a block, it is highly likely that it can also probe other IPs in the same block. Conversely, if it cannot see a certain IP, it is probably unable to see the entire block. This formalization provides a quantitative measure to validate our key insight: that IP visibility patterns exhibit strong spatial correlation within CIDR blocks, enabling efficient representative-based probing strategies. Specifically, a higher consistency score indicates greater homogeneity in intra-block visibility, robustly justifying the use of a small number of anchor IPs to accurately represent the entire block.

### Appendix C.2. Verification and Analysis

To verify the rationality of our proposed Visibility Consistency, we performed a consistency analysis on the complete data set of the probe scan from five nodes in Section 2.1. We calculated the pairwise Jaccard similarity between the visibility vectors of all IP pairs within CIDR blocks of various sizes using Equation (A3), from large /20 prefixes to small /28 prefixes, in addition to the original, highly irregular blocks from the MaxMind dataset (/Orig). The results, summarized in Figure A2, reveal a powerful and robust phenomenon:

- **High Intra-Block Consistency:** The **median** consistency score is consistently **1.00** across all block sizes. This indicates that for most blocks, knowing the visibility of one IP perfectly predicts the visibility of all others within the same block. The visibility vector of any IP is essentially interchangeable with that of its neighbors.
- **Stability Across Scales:** The **mean** consistency remains notably high (above 0.91) across all regularly-sized blocks. This demonstrates that Visibility Consistency is an inherent property driven by network routing and filtering policies, not an artifact of observation scale.
- **Existence of Outliers:** The standard deviation and the lower quartile values confirm the existence of blocks with lower internal consistency. These typically represent complex network environments such as multi-homed prefixes or blocks employing fine-grained, IP-specific filtering policies.

### Appendix C.3. Selecting the Optimal Block Size

The choice of block size is a trade-off between consistency and granularity. Table A1 shows the summary statistics table of Visibility Consistency across CIDR Block Sizes. While /Orig blocks exhibit the highest mean consistency, their irregular and often large sizes render them unsuitable for fine-grained, load-balanced task allocation. Our analysis identifies the /23 prefix as the optimal operational unit for VistaScan:

- **High Absolute Consistency:** It maintains a high mean consistency score of **0.934**, ensuring the core premise of our method holds.
- **Favorable Proportion of Difficult Blocks:** The proportion of blocks with consistency below 0.90 (**21.6%**) is among the lowest for regularly-sized splits, minimizing the potential for misrepresentation by anchors.
- **Superior Manageability:** With 8798 blocks, it provides an ample number of tasks to enable efficient load balancing across our distributed nodes, without incurring the excessive overhead of managing tens of thousands of smaller blocks.

This consistency means that probing a few anchor IPs can accurately predict a node’s visibility of the entire block. This high degree of intra-block consistency is the foundational insight that makes VistaScan efficient and practical. It allows us to accurately characterize the visibility of an entire /23 CIDR block by probing just a few representative anchor IPs within it, reducing the overhead of building the visibility matrix by orders of magnitude. For the optimal /23 block size, the median consistency score reaches 1.00 (indicating perfect consistency for most blocks), with a mean consistency score of 0.934. The 21.57% of blocks with a consistency score below 0.90 do not “completely fail to follow the consistency rule”—a Jaccard similarity of 0.90 still represents extremely high homogeneity in visibility patterns and does not invalidate the core premise of our method.

**Table A1:** Summary statistics table of Visibility Consistency across CIDR Block Sizes.

Mask	Count	Mean	Median	Std	Q1	Q3	Prop. < 0.95	Prop. < 0.90
Original	595	0.9511	1.0000	0.1211	0.9623	1.0000	0.2151	0.1361
20	2161	0.9306	1.0000	0.1248	0.9111	1.0000	0.3318	0.2272
21	3455	0.9325	1.0000	0.1268	0.9176	1.0000	0.3146	0.2272
22	5595	0.9355	1.0000	0.1231	0.9200	1.0000	0.3001	0.2193
23	8798	0.9341	1.0000	0.1281	0.9222	1.0000	0.2851	0.2157
24	12,994	0.9305	1.0000	0.1352	0.9200	1.0000	0.2872	0.2212
25	18,909	0.9252	1.0000	0.1457	0.9053	1.0000	0.2928	0.2330
26	26,194	0.9204	1.0000	0.1530	0.9000	1.0000	0.2964	0.2458
27	35,710	0.9140	1.0000	0.1632	0.8930	1.0000	0.3066	0.2529
28	48,232	0.9122	1.0000	0.1765	0.9000	1.0000	0.3001	0.2447

## Appendix D. Criteria for Anchor IP Selection

Given that a few representative anchor IPs are used to characterize an entire block, the selection of these anchors is of paramount importance. We have given this careful consideration and identified the following key factors.

- **Representativeness:** The selected IPs should be representative of their block; that is, if a node can see a given anchor IP, it should be able to see the vast majority of IPs within that block. The principle of Visibility Consistency explicitly validates the representativeness of anchor IPs. In theory, the chosen IPs should exhibit the *most prevalent visibility pattern* within the block.
- **Discriminative Power:** IPs with significant variation in visibility between different nodes should be selected. Divergent probing results for an IP from different nodes help highlight the differences in their coverage capabilities.
- **Quantity:** Due to the existence of Visibility Consistency, a single IP could, in theory, represent an entire block, offering the lowest probing and maintenance overhead. However, we opt against using just one IP for two reasons. First, a considerable proportion of blocks exhibit poor Visibility Consistency, where a single IP cannot guarantee representativeness for the whole block. Second, a single IP introduces a point of failure; if that specific IP goes offline, it could lead to the erroneous conclusion that the entire block is unreachable, thereby compromising the accuracy of all subsequent processes and the entire methodology. Hence, a *multi-anchor strategy* is necessary. The multi-anchor strategy also enables the visibility of a node to a block to be represented as a score value rather than a simple binary value. This scoring provides fine-grained differentiation, directly reflecting the node’s proficiency with the block, and tasks can be assigned based on these scores. Conversely, the number of anchors should not be excessively large, as too many would increase the probing and management overhead.

In summary, we should select IPs that demonstrate **high consensus but non-universal** visibility, those with the **most prevalent visibility pattern** of the block. Such anchors possess the most typical visibility pattern for the block, meaning that they are visible to most scanning nodes, but not all, ensuring both representativeness and discriminative power. Simultaneously, a multi-anchor strategy is adopted to enhance robustness.

Since it is impractical to pre-statistically determine the visibility pattern for every block (as it would require exhaustive probing of all blocks by all nodes), achieving optimal representativeness and discriminative power in anchor selection is challenging. As a compromise, we decided to select anchors from diverse sources to maximize the variety of their origins. Relying on a single probing source to select anchors would allow the local perspective of one node to dictate the global view of a block, introducing single-point visibility bias (preference) and consequently leading to biased selection of the best probing node. To balance representativeness, discriminative power, robustness, and system overhead, we ultimately decided to select 3 anchor IPs for each block from different probing sources. This approach significantly enhances the system’s robustness and accuracy while maintaining low overhead, laying a solid data foundation for an efficient and reliable distributed probing system.

This multi-anchor strategy from diverse probing sources aligns with established practices in network measurement, where a limited set of probes is used to infer larger-scale properties [7,12,28,33,34,35,36,37,38]. Although three anchors proved effective in our experiments, we acknowledge that the optimal number may vary with network conditions and consistency metrics. Investigating adaptive anchor selection strategies is a promising direction for future work.
